# The use of extended reality in microsurgical free flap planning – A systematic review

**DOI:** 10.1016/j.jpra.2025.04.005

**Published:** 2025-04-17

**Authors:** Kristian Havndrup Rasmussen, Magnus Balslev Avnstorp, Peter Sinkjær Kenney, Nicco Krezdorn, Michael Rose

**Affiliations:** a Department of Plastic and Breast Surgery, Zealand University Hospital, Roskilde, Denmark; b Department of Plastic and Breast Surgery, Aarhus University Hospital, Aarhus, Denmark; c Department of Clinical Sciences Malmö, Lund University, Malmö, Sweden

**Keywords:** Extended reality, Augmented reality, Virtual reality, Plastic Surgery, Microsurgery, Reconstruction

## Abstract

**Background:**

Dissecting free tissue flaps can be challenging and time-consuming. Imaging technologies are typically used to assist with preoperative planning. However, conventional methods have limitations that could potentially be overcome by extended reality technologies. This systematic review aimed to evaluate the current use of extended reality (XR) in preoperative planning for microsurgical free flaps and assess its effectiveness as a tool in this context.

**Methods:**

The systematic review was conducted following the preferred reporting items of systematic reviews and meta-analyses guidelines. Articles identified through the PubMed, Ovid, Web of Science, and Scopus databases were screened based on titles and abstracts, and relevant articles were assessed in full text. Inclusion criteria were: use of XR for vascular visualization in plastic and reconstructive surgery, reported workflow, accuracy, operative times, the surgeon’s opinion, or complications. Exclusion criteria were: unavailability of full text, non-English, non-human, or other review articles. Quality assessment was performed using center for evidence-based medicine level of evidence and Joanna Briggs Institute critical appraisal tools.

**Results:**

Overall, 7 articles on virtual reality and 26 articles on augmented reality in plastic and reconstructive surgical procedures were identified. Virtual reality demonstrated better visualization of patient-specific anatomy and was more time efficient than CT angiography. Augmented reality was found to be more accurate and time efficient than Doppler ultrasound. However, successful XR implementation necessitates a well-designed workflow. The results are limited by the designs of the included studies and level of evidence they represent.

**Conclusion:**

XR technologies show promise in preoperative planning of microsurgical free flaps. Standardized outcome measurements and prospective, comparative studies are essential for a comprehensive understanding of the clinical value of XR-based preoperative planning.

## Introduction

In microsurgical reconstruction, meticulous dissection of the perforator vessels supplying the flap tissue is crucial. The dissection of these vessels can be difficult and time-consuming.^1^ If executed improperly, damage to the vessels could result in flap loss or other complications such as partial necrosis or hematoma. Additionally, it is important to account for the variations in the vascular anatomy when raising free flaps such as a deep inferior epigastric artery perforator (DIEP).^2^ Identifying and localizing relevant perforators is crucial in optimizing flap survival and minimizing complication rates and procedure times.

Different techniques have been used for preoperative planning of free flaps, including Doppler ultrasound,^3^^,^^4^ color duplex scanning,^5^ and computed tomography angiography (CTA),^6^^,^^7^ the latter is generally considered the gold standard. Additionally, 3D rendered CTAs can be used.^8^ Magnetic resonance angiography (MRA) has also been used for perforator identification with accurate results.^9^ Non-imaging methods evaluating target perforators intraoperatively has also been proposed.^10^ However, conventional imaging methods continue to have some limitations, including the transfer of information from modalities, such as CTA or MRA to the patient, and lack of opportunities for intraoperative navigation. New methods to overcome these limitations could possibly improve preoperative planning, intraoperative guidance, and surgical outcomes, including operative time and complication rates.

Extended reality (XR) refers to technologies capable of combining real-world objects with digital objects to varying degrees. XR encompasses virtual reality (VR), a completely virtual environment, and augmented reality (AR), a mixed real environment with digitally overlayed images. XR images can be generated from data obtained from CTA or MRA, providing surgeons with novel means of interacting with the data.

XR technologies have shown promise as tools for preoperative planning and intraoperative navigation within several surgical specialties such as orthopedic hip surgery, spine surgery, urology, and neurosurgery.^11^, ^12^, ^13^, ^14^

In this systematic review, we aimed to examine the utilization of XR technologies in the context of perforator localization within plastic and reconstructive surgical procedures. We sought to address its performance compared to other methods with respect to aspects such as workflow, accuracy, operative time, and complications.

## Methods

This systematic review was authored in accordance with the preferred reporting items of systematic reviews and meta-analyses (PRISMA) guidelines.^15^

### Research question

This systematic review sought to answer the following research question:

“In the context of perforator identification in plastic and reconstructive free flap surgery, how does the utilization of extended reality compare to other non-extended reality technologies regarding user experience and clinical outcomes?”

### Systematic literature search

A systematic literature search was conducted by the primary author, KHR, on October 18, 2023 and updated on December 1, 2023 and October 18, 2024. The literature search was designed around the research question using 2 search blocks, incorporating the search terms related to XR technologies and plastic and reconstructive free flap surgery. These 2 search blocks were linked with the Boolean operator “AND.” Details of the systematic literature search are provided in Supplementary Information 1.

The systematic literature search was performed across 4 databases: PubMed, Ovid, Web of Science and Scopus. Subsequently, a de-duplication process was carried out using EndNote 20, following the methodology described by Bramer et al.^16^. The resultant articles were manually and critically appraised by the author, KHR, using the Covidence screening tool (Covidence systematic review software, Veritas Health Innovation, Melbourne, Australia. Available at www.covidence.org). Initially, the articles were screened based on titles and abstract, and relevant articles were retrieved and assessed based on the full text.

Inclusion criteria for the studies were: Utilization of XR for vascular visualization, plastic and reconstructive surgical procedures or simulation of one, report on workflow, accuracy, preoperative or intraoperative time, and surgeons’ opinion of XR or complications. Articles not available in full text, not written in English, non-human studies, or review articles were excluded.

### Data extraction

Data extracted by the primary author, KHR, included title, authors, year of publication, study design, XR technique, imaging modality, the setting in which XR is applied, population size, flap type, and outcomes included accuracy of examined XR modality, postoperative complications, preoperative or intraoperative times, workflow, and surgeons’ opinions on the technology.

This review included studies that lacked quantitative data but instead provided descriptive accounts of the workflow related to the implementation of XR technologies in plastic and reconstructive flap surgery. Although these studies lack data, they were included to offer insights into the process of incorporating these technologies in surgical procedures.

### Synthesis

The included studies were grouped depending on the type of XR modality used. Among the 33 included studies, 7 used VR and 26 used AR. Outcome data were standardized and compared. Outcome data are presented in Table 1, Table 2.

**Table 1:** Characteristics of the included studies in the VR group

Author(s)	Imaging modality	Study design	Setting	Population size	Procedure	CEBM level	Outcome	JBI score
P. Gacto, et al.^20^	CTA	Case report	Preoperative planning	n = 1	ALT flap	5	Total match between VR visualized dominant perforator and intraoperative findings 0 postoperative complications	5/8
Gacto-Sanchez, et al.^21^	CTA	Cohort study (consecutive patients)	Preoperative planning	n = 12	DIEP flap	3	100% correlation between preoperatively selected perforator and intraoperative findings Error of VR perforator localization compared with intraoperative findings: Mean error = 0.23 cm (95% CI, 0.17-0.30) Perforator <4 cm from the umbilicus: Mean error = 0.17 cm (95% CI, 0.09-0.024) Perforator >4 cm from the umbilicus: Mean error = 0.31 cm (95% CI, 0.21-0.42)	5/11
C. Suárez-Mejías, et al.^22^	CTA	Cohort study	Preoperative planning	n = 35 (consecutive)	DIEP flap	3	Mean operative time CTA-VirSSPA = 478 min (SD 56.94) Control (Doppler) = 606.29 min (SD 81.94) (p < 0.001) Flap related postoperative complications CTA-VirSSPA = 0 Control = 14 (p < 0.001) Donor site complications CTA-VirSSPA = 2 Control = 15 (p = 0.001)	7/11
T. Suffee, et al.^23^	CTA	Case series (prospective)	Preoperative planning	n = 30 (33 flaps), consecutive	DIEP flap	4	Perforator chosen by radiologist (CTA): 16/30 (53%) Perforator chosen by surgeon (VR): 10/30 (33%) Concordance of best perforator between VR and CTA = 33 %	8/10
G. De la Cruz-Ku, et al.^26^	MRI	Case report	Preoperative planning	n = 1	NSM + Recon-struction	5	3 perforators identified in the breast preoperatively, all of these were conserved during surgery	6/8
D. Freidin, et al.^25^	CTA	Case series (prospective)	Preoperative planning	n = 30 (42 flaps)	DIEP flap	4	Surgeons’ rating of VR vs. CTA in preoperative planning Modified surgical plan after VR assessment in 4/42 flaps, no complications were reported Flap loss in 1/42 flaps Minor complications in 6/42 flaps	6/10
A. Di Via Ioschpe, et al.^24^	CTA	Retrospective case series	Preoperative planning	n = 44	DIEP flap, SIEA flap	4	Pearson correlation between VR and CTA based on distance of perforator from umbilicus: Unilateral perforator: R = 0.960, p < 0.001 Bilateral perforators: R = 0.930, p <0.001 Time spent on interpretation of images: CTA: Average 60 min VR (first 10 patients): 37 min VR (next 20 patients): 9.3 min	7/10

Abbreviations: ALT, anterolateral thigh; CTA, computed tomography angiography; DIEP, deep inferior epigastric artery perforator; NSM, nipple-sparing mastectomy; SIEA, superficial inferior epigastric artery; VR, virtual reality

**Table 2:** Characteristics of the included studies in the AR group

Reference	AR type	Imaging modality	Study design	Setting	Population size	Procedure	CEBM level	Outcome	JBI score
S. Battaglia, et al.^27^	Tablet	CTA	Case series (consecutive)	Preoperative planning/intraoperative navigation	n = 3	FF flap	4	Description of workflow	3/10
S. Battaglia, et al.^28^	Tablet	CT	Case series	Preoperative planning	n = 8	Pedicled flap	4	0 flap-related postoperative complications 1 non-flap-related postoperative complication (temporary paralysis of facial nerve)	4/10
A. Fitoussi, et al.^31^	Tablet	CT	Proof of concept	Preoperative planning	n = 12 (26 perforators)	DIEP flap	5	Median distance between AR and Doppler markings 2 mm Median distance between AR and CTA markings: 2.5 mm 92% of the AR markings were ≤1 cm from Doppler marking No total flap loss or partial necrosis	
N. Pereira, et al.^41^	Smartphone	CTA	Cohort study	Preoperative planning	AR group: n = 30 (60 inguinal areas) Non-AR group: n = 15	SCIP flap	3	100% correlation between AR and Doppler markings for DIEP, sSCIP and dSCIP 100% correlation with AR markings with intraoperative findings (30/30) Average flap harvest time reduced by 20% from 90 min (Doppler) to 72 min (AR)	6/11
N. Pereira, et al.^42^	Smartphone	CTA	Prospective case series	Preoperative planning	n = 89 (101 flaps)	SCIP flap	4	Postoperative complications after implementing AR to SCIP flap: 9/101 revision surgeries 6/101 flap losses 12/101 partial flap losses 2/101 wound dehiscence 1/101 hematoma 1/101 seroma	8/10
N. Pereira, et al.^47^	Smartphone	CTA	Proof of concept	Preoperative planning	n = 15 (30 thighs/groins)	SCIP flap	5	AR images showed 100% correlation to Doppler ultrasound	
S. Hummelink, et al.^32^	Projector	CTA	Technical report	Preoperative planning	n = 9	DIEP flap	5	Audible Doppler signal at AR marking in 98/100 Correctly identified perforators 84.3% ± 25.8% (AR) vs. 56.9% ± 31.4% (Doppler) (p = 0.030) Average distance from marking to intraoperative findings: 4 mm ± 3 mm (AR) vs. 7 mm ± 4 mm (Doppler; p = 0.009)	
S. Hummelink, et al.^34^	Projector	CTA	Case series	Preoperative planning	n = 10	DIEP flap	4	Description of workflow	7/10
S. Hummelink, et al.^35^	Projector	CTA	Proof of concept	Preoperative planning	n = 6 (9 breasts)	DIEP flap	5	Audible Doppler signal at AR marking in 41/42 0 complications	
M. P. Chae, et al.^29^	Projector	CTA	Case series	Preoperative planning	n = 6	ALT flap^3^, TUG flap^1^, DIEP flap^2^	4	Description of workflow	2/10
I. J. Cifuentes, et al.^51^	Projector	DIRT	Proof of concept	Preoperative planning	n = 3 (6 thighs)	ALT flap	5	100% correlation of AR projection and Doppler signal Time needed to identify perforators via AR 3.5 min (range 3.3-4.0 min)	
S. Hummelink, et al.^33^	Projector	CTA	RCT	Preoperative planning	n = 49 (69 flaps) Projection: n = 28 (40 flaps) Doppler: n = 21 (29 flaps)	DIEP flap	2	Mean preoperative perforator localization time: 2.3 ± 0.8 (AR) vs. 20 ± 5.5 min (Doppler, p = 0.001) Correctly identified perforators: 61.7 ± 7.3 (AR) vs. 41.2 ± 8.2 (Doppler, p = 0.020) Flap harvest time: 136 ± 7 (AR) vs. 155 ± 7 (Doppler, p = 0.012) No significant difference in complications between the 2 groups	9/13
J. Martschinke, et al.^38^	Projector	CTA	Pilot study	Preoperative planning	n = 10	DIEP flap	5	Description of workflow	
Y. F. Tang, et al.^44^	Projector	CTA	Prospective cohort	Preoperative planning	n = 14	ALT flap	3	Accuracy of AR projection vs. Doppler 90.2% (37/41) vs. 82.4% 28/34) Sensitivity of AR projection vs. Doppler 97.4% (37/38) vs. 73.7% (28/38) Complications: 1 patient with infection, 1 patient with partial flap necrosis	4/11
N. Yodrabum, et al.^46^	Projector, smartphone	CTA	Case series	Preoperative planning	n = 8	FF flap	4	Average error of AR projection vs. Doppler 0.7 ± 0.2 cm	3/10
E. L. Meier, et al.^52^	Projector	DIRT	Proof of concept	Preoperative planning	n = 50 (71 flaps)	DIEP flap	5	Description of workflow DIRT vs. CTA compared with intraoperative findings: 56.8% (DIRT) vs. 59.8% (CTA) of perforators visualized by imaging corresponded to an intraoperatively located perforator 54.7% of direct matches between DIRT and intraoperative perforators appeared within the first 5 emerging hotspots Total match between DIRT-based AR, Doppler, CTA, and intraoperative findings in 50.8% of perforators; 62.7% of these emerged within the first 5 DIRT hotspots 47.5% of all DIRT hotspots were directly related to a CTA-images perforator 27.0% of all DIRT hotspots were related to SIEV/SIEA vessels 25.5% of DIRT hotspots were unexplained	
M. Katayama, et al.^37^	HMD	CTA	Case reports	Preoperative planning	n = 2	Lower extremity reconstruction	5	Difference in depth perception between 2 types of holograms	3/8
T. R. Jiang, et al.^36^	HMD	CTA	Technical report	Experimental	n = 7	Simulation	5	Accuracy in illuminated vs. non-illuminated conditions: 2.85 mm vs. 1.35 mm, P = 0.02 No significant difference between accuracy in different light intensities No significant difference in accuracy induced by head rotation or walking	
T. S. Wesselius, et al.^45^	HMD	CTA	Proof of concept	Preoperative planning	n = 1	DIEP flap	5	Description of workflow	
T. Nuri, et al.^40^	HMD	CTA	Case report	Preoperative planning	n = 1	ALT flap	5	10 min for alignment of AR overlay 1 cm difference between AR and Doppler markings AR markings corresponded perfectly with intraoperative findings 0 postoperative complications	4/8
I. Seth, et al.^43^	HMD	CTA	Case series	Preoperative planning/ intraoperative navigation	n = 5	DIEP flap	4	Description of workflow Time spent converting CTA to AR-usable file = approx. 60 min per case Image registration = 7-10 min Perforator markings = 5-7 min Perfect match between AR and Doppler ultrasound in 4/5 cases In the 1 case with misalignment, CTA was 16 months old and the patient had lost weight	5/10
M. F. Berger, et al.^50^	HMD	MRA	Pilot study	Preoperative planning	n = 10	DIEP flap	5	Description of workflow SUS = 67, qualifying as "moderate to good"	
G. Masterton, et al.^39^	HMD	CTA	Proof of concept	Preoperative planning	n = 2	DIEP flap	5	Description of workflow	
D. J. Cholok, et al.^30^	HMD, simulated	CTA	Technical report	Experimental	n = 19	Simulation	5	Mean difference between naked eye visualization and HMD visualization for 2 points was 9.9 mm (SE 0.106) and 13.7 mm (SE 0.106)	
F. N. Necker, et al.^49^	HMD	CTA	Proof of concept	Preoperative planning	n = 4	DIEP flap	5	Through AR and HMDs, 2 surgeons can be in a shared physical-virtual space and discuss the relevant anatomy and DIEP flap harvest while being at different locations	
Y. Liu, et al.^48^	HMD	CTA	Experimental	Preoperative planning	n = 40 (20 experimental and 20 control)	ALT and fibula osteocutaneous flaps	2	No statistically significant difference in operative time. Significantly less average blood loss in the AR group compared with the CDU group (24 mL vs. 56 mL). Slightly less recognition rate of perforators in the AR group vs. the CDU group (93.9% vs. 97.2%), non-significant	

Abbreviations: ALT, anterolateral thigh; CDU, color Doppler ultrasound; CTA, computed tomography angiography; DIEP, deep inferior epigastric artery perforator; DIRT, dynamic infrared thermography; FF, free fibula; HMD, head-mounted display; MRA, magnetic resonance angiography; RCT, randomized controlled trial; SCIP, superficial circumflex iliac artery perforator; SE, standard error; SIEA/SIEV, superficial inferior epigastric artery/vein; SUS, system usability assessment; TUG, transverse upper gracilis

### Quality assessment

All articles were assigned a center for evidence-based medicine (CEBM) level of evidence.^17^ Because of the limited research on XR technologies in plastic and reconstructive surgery, no articles were excluded based on a low CEBM level of evidence. However, readers should consider the low level of evidence of some of the included studies.

Quality assessments were performed using the Joanna Briggs Institute (JBI) Critical Appraisal Tools.

## Results

### Study selection

**Figure 1:**
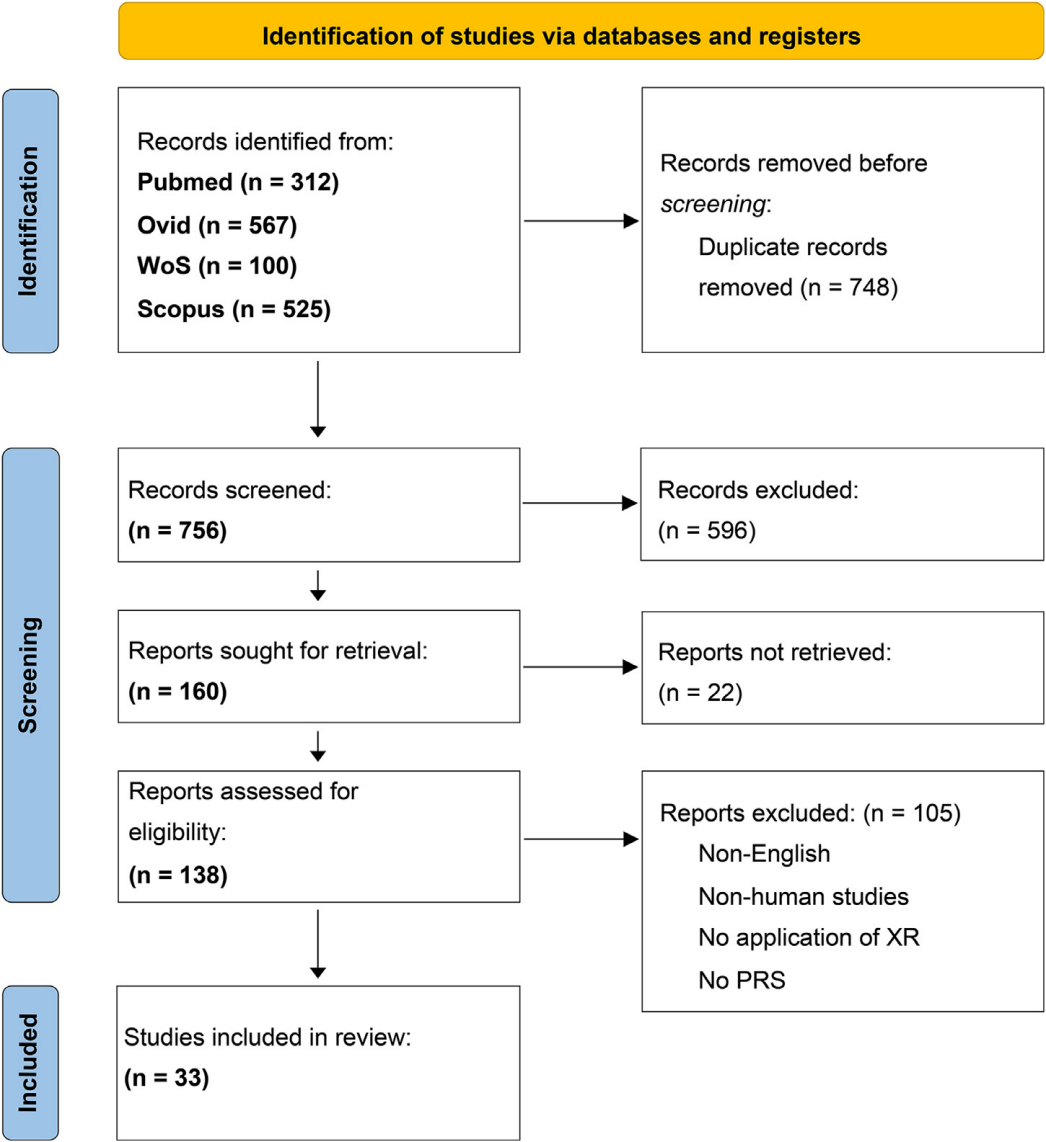
PRISMA 2020 flowchart*. Abbreviations: 3D = three-dimensional, PRS = plastic or reconstructive surgery, WoS = Web of Science, XR = extended reality*

The systematic literature search identified 756 articles after de-duplication. After title/abstract and full-text screening, 33 articles met the eligibility criteria and were included in the systematic review. Two articles^18^^,^^19^ were excluded as they reported on studies that were covered by 2 other articles. An overview of the systematic literature search is presented in the PRISMA flowchart in Figure 1. Details about the characteristics of the included studies can be found in Table 1, Table 2.

### Quality assessment

#### CEBM level of evidence

In the VR group, 2 studies ranked as level 3, 3 studies ranked as level 4, and 2 studies ranked as level 5. In the AR group, 2 studies ranked as level 2, 2 studies ranked as level 3, 7 studies ranked as level 4, and 12 studies ranked as level 5.

#### JBI Critical Appraisal Tools

In the VR group, the cohort studies scored a mean of 6/11 (range 5-7), case series scored a mean of 7/10 (range 6-8), and case reports scored a mean of 5.5/8 (range 5-6). In the AR group, randomized controlled trials (RCTs) scored 9/13, cohort studies scored a mean of 5/11 (range 4-6), case series scored a mean of 4.6/10 (range 2-8), and case reports scored a mean of 3.5/8 (range 3-4).

Some studies were not critically appraised using the JBI Critical Appraisal Tools, as they were designed as proof of concept studies or technical reports; hence, they were included to provide insights into the implementation of XR technologies in plastic and reconstructive surgery.

### Workflow of XR-assisted preoperative planning

**Figure 2:**
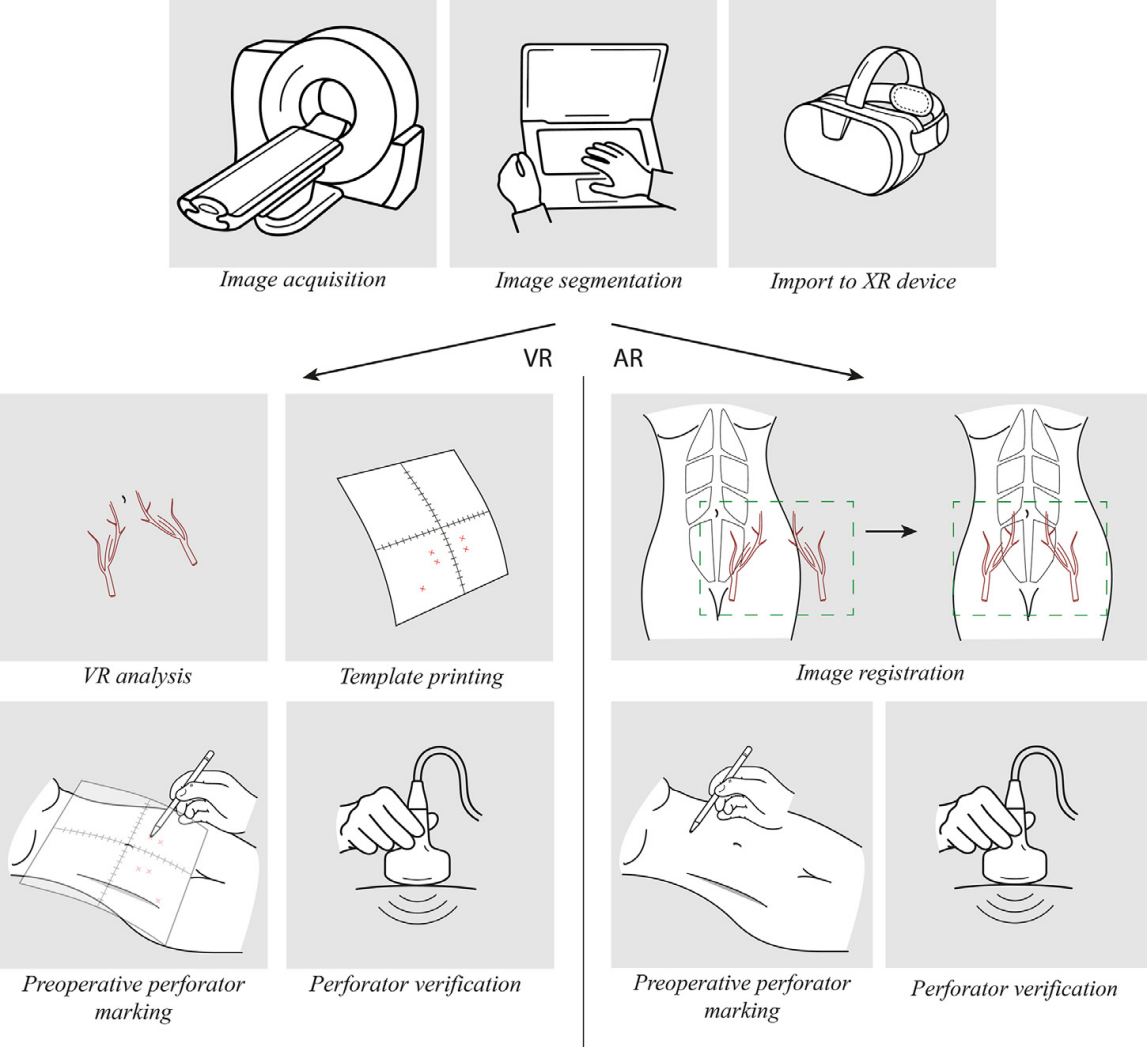
Overview of the workflow of XR-based preoperative planning

The workflow of incorporating XR technologies in preoperative planning for microsurgical reconstructive procedures are described in several studies. For VR and AR, some of the steps described below are identical, whereas others differ between the technologies. Moreover, differences in the workflow within VR and AR are described in the literature. A generalized workflow will be introduced below and is illustrated in Figure 2

1. Image acquisition

For VR and AR, image data need to be acquired for processing. In 6 of the included studies on VR, images were based on CTA data.^20^, ^21^, ^22^, ^23^, ^24^, ^25^ In one VR study, MRI was used.^26^ In the AR studies, CTA was the most used,^27^, ^28^, ^29^, ^30^, ^31^, ^32^, ^33^, ^34^, ^35^, ^36^, ^37^, ^38^, ^39^, ^40^, ^41^, ^42^, ^43^, ^44^, ^45^, ^46^, ^47^, ^48^, ^49^ while MRA was used in 1 study^50^ and direct infrared thermography (DIRT) was used in 2 studies.^51^^,^^52^ DIRT uses thermal cameras to generate color-coded temperature maps of the tissues, enabling the visualization of perforators, which appear as hotspots in the image.^52^

When using AR, anatomical landmarks must be identified for later alignment of AR image with the patient (see step 4). Skin landmarks with adhered radiopaque markers have been suggested.^45^ Other studies suggest landmarks such as the xiphoid process, umbilicus, anterior superior iliac spinae, or superior border of the pubic symphysis.^33^^,^^41^^,^^50^ Additionally, oil fiducials markers, which are glues with oil that can be visualized on MRA, has been suggested as landmarks when using MRA-based AR.^50^

One study obtained a 3D surface scan of the abdomen to account for soft-tissue deformation, which may improve the accuracy of the AR system.^50^ In 1 case, a splint was made to fix the extremity in a certain position during scanning and surgery, minimizing the shift between AR overlay and upper extremity position during surgery.^37^

2. Image segmentation

Image data are then imported into computer software for processing. Tissues are segmented to facilitate good visualization of the vessels of interest.^22^^,^^25^ The 3D images made through image segmentation are then imported into other types of software such as Blender for manipulation, in which color and transparency can be added or adjusted.^37^^,^^43^

3. Import to XR device

**Figure 3:**
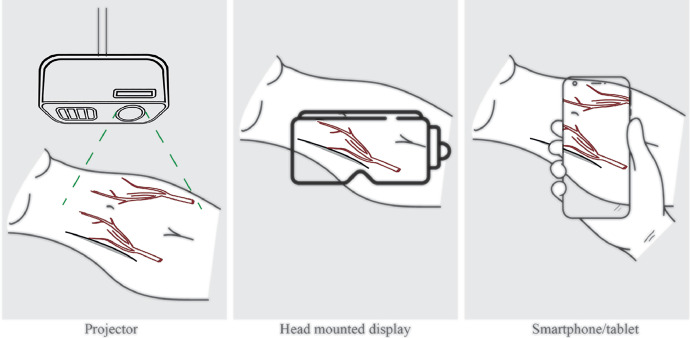
Projector-based AR, HMD-based AR, and smartphone/tablet-based AR

The 3D image file is then imported into the chosen XR device, which for VR can be either a computer or VR headset, and for AR, it can be either a computer with a projector, smartphone/tablet, or head-mounted display (HMD), which is a glasses-like device with a see-through display capable of superimposing digital data onto the real world (Figure 3).

#### VR workflow

4. VR image analysis

VR images are then observed and analyzed by the surgeon. In early VR, these images were viewed on a computer screen.^20^, ^21^, ^22^, ^23^ In more recent VR, the images are imported into VR 3D glasses, the surgeon uses these glasses to view and analyze the images.^24^^,^^25^ The VR images can be viewed from different angles, which allows localization and measurement of the vessels of interest.^21^^,^^24^, ^25^, ^26^ Thus, the most suitable perforator for flap harvest can be determined.^23^

5. Transfer of anatomical information to patient and preoperative perforator marking

After measurement is carried out in the VR setting, they can be printed as a template scale, which is then transferred to the patient and the anatomy is traced onto the patient.^21^^,^^23^ Suffee et al. suggested to verify the appearance of a vessel using Doppler ultrasound.^23^

#### AR workflow

4. Image registration

The registration process ensures that the overlay of the 3D images corresponds accurately to the real-world anatomy. This alignment is typically performed manually,^43^^,^^50^ although automatic methods of alignment have been reported.^34^^,^^38^^,^^52^ This should ideally facilitate complete matching of the superimposed image-based anatomy and real-world anatomy in orientation and size, thereby enabling the initiation of flap preparation.^45^ Previously identified anatomical landmarks (step 1) are located^40^^,^^45^^,^^50^ using sterilized stainless-steel pointers with a QR marker for HMDs.^45^ An alternative registration method where the scale of the 3D model is adjusted to match the patient using the umbilicus as a reference point has also been proposed.^39^

5. Preoperative perforator marking

Following registration, the linkage of the holographic anatomy to the patient eliminates the necessity for visible anatomical landmarks, enabling the surgeon to initiate preoperative planning guided by the holographic overlay of the vascular anatomy.^45^ If relevant to the surgery, other structures such as lymph nodes can also be displayed, as suggested by Hummelink et al.^34^. Besides using the digital overlay of the vascular anatomy for preoperative planning, some studies also suggest using it intraoperatively for guiding the dissection.^27^^,^^43^

One study proposed a method to verify the presence of a perforator at the AR-based perforator marking by examining the area using a Doppler ultrasound unit.^39^

### Virtual reality

Seven studies (24%) investigated VR for preoperative planning of microsurgical flaps.^20^, ^21^, ^22^, ^23^, ^24^, ^25^, ^26^ Outcome data are presented in Table 1.

#### Accuracy of VR-assisted preoperative planning

Four studies evaluated the accuracy of VR for preoperative perforator identification.

In determining “the most suitable perforator” for DIEP flaps through preoperative imaging, Suffee et al. found VR to be inferior to CTA.^23^ Contrastingly, Di Via Ioschpe et al., in a retrospective manner, compared perforator visualization using VR with visualization using CTA and reported a strong correlation between the 2 methods when examining three-dimensional distances.^24^ The mean error of VR-assisted perforator markings compared to intraoperative findings has been reported to be 0.23 cm, and significantly less for perforators located closer than 4 cm to the umbilicus.^21^ A case report demonstrated perfect alignment between preoperative perforator markings and intraoperative findings,^20^ and another case report identified and conserved all preoperatively visualized perforators in the breast.^26^

#### Preoperative and intraoperative use of time

Two studies evaluated either preoperative or intraoperative times used in relation to VR for microsurgical reconstruction.

Using VR for preoperative planning of DIEP flap-based breast reconstruction, Suarez-Mejias et al. observed a 21% reduction in total operative time, from 606 to 478 min, compared with Doppler ultrasound-based preoperative planning.^22^ In addition, Di Via Ioschpe et al. observed a reduction in the time spent to analyze preoperative imaging for 10 patients undergoing abdominal-based free flap, from 60 min using CTA to 37 min using VR. The time used to analyze VR imaging was lesser for the subsequent 20 patients.^24^

#### Postoperative complications

Four studies evaluated the rate of postoperative complications following VR-assisted free flap surgery. In one study, VR-assisted DIEP flap planning resulted in significantly less postoperative complications related to the flaps and donor-sites compared to Doppler ultrasound planning, although the type of complications were not specified.^22^ In other studies, postoperative complications were addressed but failed to provide comparative results to other methods.^20^^,^^23^^,^^25^

#### Surgeons’ opinions

Freidin et al. compared VR to CTA for preoperative planning of abdominal free flaps for breast reconstruction. A questionnaire based on a 1-5 Likert scale was used. They concluded that in 96.55% of the 30 cases, the surgeons rated VR to provide new information. Overall, 72.4% of the surgeons rated VR to be superior to CTA in terms of similarity to reality. However, in most cases, the surgical plan remained unchanged when visualizing the perforators using VR, suggesting that CTA is a more reliable method for preoperative planning.^37^

### Augmented reality

Twenty-six studies investigated AR for preoperative planning of microsurgical flaps.^27^, ^28^, ^29^, ^30^, ^31^, ^32^, ^33^, ^34^, ^35^, ^36^, ^37^, ^38^, ^39^, ^40^, ^41^, ^42^, ^43^, ^44^, ^45^, ^46^^,^^50^^,^^51^ Outcome data are presented in Table 2.

#### Accuracy of AR-assisted preoperative planning

Fifteen studies evaluated the accuracy of AR for perforator localization. In general, the accuracy is reported in comparison with either conventional perforator visualization methods or with intraoperative findings (measurements relative to umbilicus).

Five studies compared preoperative AR markings of perforator localization with Doppler markings.^30^^,^^31^^,^^40^^,^^46^^,^^50^ Among these studies, the distance between AR-markings and Doppler markings ranged from 2 to 17.7 mm.

Three studies compared preoperative AR markings with intraoperative measurements of perforator localization in relation to the umbilicus.^32^^,^^41^^,^^44^ The mean error in accuracy of these studies ranged from perfect correlation to 5.3 mm error. One study compared these results to Doppler markings, and reported greater accuracy of AR compared to Doppler (5.3 mm vs. 19.7 mm).^44^

Three studies examined the ability of AR to detect the presence of perforators and compared this to handheld Doppler,^32^^,^^33^^,^^44^ demonstrating greater sensitivity for perforator localization of AR compared to handheld Doppler. In contrast, one study reported slightly less recognition rate of perforators by AR compared with color Doppler ultrasound (93.9% vs. 97.2%), although these results were non-significant.^48^

Eight studies evaluated the accuracy of AR-based perforator localization by using Doppler ultrasound on AR-based markings, thus in theory confirming the presence of a perforator at this marking.^29^^,^^31^^,^^32^^,^^35^^,^^41^^,^^43^^,^^47^^,^^51^ In 7 of the studies, Doppler signal was positive in 92% to 100% of the perforators. In a case series involving 5 patients, perfect match between AR markings and Doppler signal was found in 4 out of 5 cases. In the case where perfect match was not achieved, AR images based on a 16-month-old CTA were used. During this period, the patient’s weight distribution changed, resulting in a 10 mm difference between AR marking and Doppler ultrasound.^43^

A study evaluated the accuracy of HMD-based AR perforator localization under different lighting conditions, various visual angles, and movement-induced errors in a simulated perforator localization setting.^36^ No significant difference in accuracy based on the visual angle, light intensity, and no movement-induced errors was observed. When comparing localization accuracy under illuminated (specifically at low intensity) and non-illuminated conditions, a significant difference in the mean error was found (2.85 mm vs. 1.35 mm, P = 0.02).^36^ In addition, they found HMD-based AR to be accurate with a minimum mean error of 1.35 mm and maximum error of 3.18 mm.^36^

#### Preoperative and intraoperative use of time

Nine studies reported on the influence of AR on preoperative planning time or intraoperative time.

Six studies evaluated additional time required to integrate the AR system into their workflow.^29^^,^^33^^,^^39^^,^^40^^,^^43^^,^^50^ Time required to generate 3D images was reported to range from 10^33^^,^^50^ and 15 min^39^ to 60 min.^43^ Image registration was completed between 7 and 11 min^40^^,^^43^^,^^50^; however, one study reported nearly instantaneous registration, which prompted them to abandon plans to measure its duration.^29^

Three studies reported on the time spent on preoperative perforator localization. In an RCT, the use of AR significantly reduced the time spent on preoperative planning compared with the Doppler ultrasound (2.3 min vs. 20 min).^33^ Other studies reported mean times spent on perforator localization using AR from 3.5 to 7 min.^43^^,^^51^

Two studies evaluated the impact of using AR for preoperative planning on intraoperative time. Hummelink et al. demonstrated a significant reduction in flap harvest time by 12%, from 155 to 136 min, when comparing Doppler to AR.^33^ Similarly, Pereira et al. reported a reduction in flap harvest time by 20%, from 90 to 72 min, when using a smartphone-based AR perforator localization app.^41^ In contrast, Liu et al. observed no significant difference in operative time when using AR or color Doppler ultrasound for preoperative planning of ALT or free fibula flaps.^48^

#### Postoperative complications

Eight studies reported on the complications following AR-based preoperative planning.

In an RCT, Hummelink et al. found no significant difference in flap-related nor donor site-related complications between an AR and a Doppler group.^33^ Liu et al. observed significantly less average blood loss when using HMD-based AR for preoperative planning of ALT and free fibula flaps, compared to color Doppler ultrasound (24 mL vs. 56 mL).^48^ Six other studies, primarily case series, reported the rates of complications and by the nature of their studies, they precluded direct comparison with other imaging modalities.^28^^,^^31^^,^^35^^,^^40^^,^^42^^,^^44^

#### Surgeons’ opinions

One study assessed feasibility of AR use for surgical flaps through questionnaires, using the System Usability Scale based on a 1-5 Likert scale. They reported a mean SUS score of 67, classifying the system as “moderate to good” in a classification system proposed by the authors.^50^ However, experienced surgeons rated the AR system as a “nice, but not necessary tool for clinical practice”.^50^

## Discussion

XR technologies are emerging in the medical field. With their abilities to provide new representations of CT and MR scans, they open up new possibilities in the preoperative and intraoperative phase of microsurgical free flap procedures. We performed a systematic review to examine the utilization of XR in perforator localization and its comparison with other imaging modalities in terms of workflow, user experience, and clinical outcomes.

From this review, we found that XR technologies, VR and AR, show promise in the field of plastic and reconstructive surgery. Both technologies were found to be capable of reducing the time spent on preoperative perforator mapping and total intraoperative time compared with CTA and Doppler, respectively.^22^^,^^24^^,^^33^^,^^41^ Reducing preoperative times may be beneficial for surgical departments and patients, although it could be argued that preoperative perforator marking could be done, for example, a day in advance of the surgery, thereby not influencing the surgical capacity. Reducing intraoperative times may be beneficial in a clinical setting by increasing the surgical capacity and reducing patient complication rates by reducing anesthesia time. However, “free-style” free flap techniques, in which no technologies are used preoperatively to identify perforators, are also proposed as time-reducing without increasing the complication rates.^53^ This could limit the necessity for developing new technologies to aid perforator localization, although it could be argued that some surgeons, especially the less experienced surgeons, may continue to benefit from technologies such as XR for these complicated procedures.

AR shows promise in improving perforator localization accuracy, demonstrating greater accuracy when compared with Doppler.^32^^,^^33^^,^^44^ However, CTA is considered the gold standard for perforator mapping. Therefore, future research should focus on comparing CTA with and without XR technology. Regarding perforator localization accuracy, one source of error may be the difference between the anatomy visualized through AR (based on e.g., CTA) and actual anatomy at the time of surgery. Seth et al. reported that in one case, a small weight distribution change in the abdomen led to a change in perforator localization between AR-visualized CTA-based anatomy and the intraoperative anatomy (16 months post CTA).^43^ However, using DIRT as an imaging modality for e.g., projector-based AR may overcome this problem by projecting real-time anatomy onto the patient, thereby possibly improving perforator localization accuracy.^52^

However, the results from the studies on VR perforator localization accuracy are contradictory. Therefore, more studies are needed to make any conclusions.

One study found that VR had the potential to lower complication rates when compared with Doppler,^22^ and another study found no difference in complication rates when using AR instead of Doppler.^33^ However, most studies did not include comparisons with other modalities; therefore, the impact of these technologies on complication rates is still unknown.

User experience was assessed in a VR study and another AR study. VR has been proved to provide the surgeons with more realistic anatomical information,^37^ and AR was scored as a moderate to good system, although experienced surgeons rated the system as “nice, but not necessary for clinical practice”,^50^ which could indicate that they considered conventional methods to be sufficient for preoperative planning. An interesting aspect is whether XR technologies are more beneficial for less experienced surgeons, which should be examined in future studies.

When using AR for perforator identification, surgeons can realistically assess digital representations of the anatomy and real anatomy simultaneously, which may be beneficial in preoperative planning and intraoperative navigation, as suggested in 2 studies.^27^^,^^43^ However, this is not the case when using VR for perforator identification, as the surgeon will be in a completely digital environment that is separate from the actual patient. Interestingly, no studies on perforator identification have yet compared VR to AR; hence, the benefits of bridging the gap between the digital and real world is still unknown. Additionally, the quality of the radiologic dataset used for 3D rendering may also influence the quality of the XR images, which could be a limiting factor for these technologies. Determining optimal scanning protocols to generate adequate XR images should be a focus for future research.

The literature on XR use in plastic and reconstructive microsurgery is limited by the quality assessment findings in this systematic review, indicating risk of bias and low-quality studies with low level of evidence. Therefore, high-quality, comparative, and prospective studies are needed to fully determine the clinical value of these technologies in plastic and reconstructive surgical procedures. Furthermore, the studies are limited by the lack of objective measures on different clinical outcomes. Therefore, we suggest that future studies on this topic should standardize outcome measures including preoperative time spent on perforator mapping, intraoperative times, accuracy of perforator localization measured as preoperative markings compared with intraoperative localization of the perforator, and postoperative complications.

## Conclusion

Extended reality technologies, including VR and AR, show promise as tools for preoperative perforator localization in plastic and reconstructive surgery. These systems can realistically present patient-specific anatomy to the surgeon and potentially offer greater time efficiency compared to conventional imaging modalities. However, the clinical value of XR in this field remains uncertain due to the limitations in the current literature, including potential biases and low levels of evidence. Therefore, we recommend conducting further high-quality studies with standardized and objective outcome measures.

## Conflicts of interest

PSK was co-author on one of the included articles in this review.

KHR, MBA, NK, and MR declare that they have no competing interests associated with the manuscript.

## Appendix Supplementary materials

Image, application 1

Image, application 2

Image, application 3
