# Supplementary material for: The use of extended reality in microsurgical free flap planning – A systematic review
Source: JPRAS Open. 2025 Apr 17;45:1–19. doi: 10.1016/j.jpra.2025.04.005 (PMC12149546; doi:10.1016/j.jpra.2025.04.005)
Supplement: Supplementary file 1 [file mmc1.docx]

## Literature search terms

**Pubmed:**

("Augmented Reality"[Mesh] OR "Virtual Reality"[Mesh] OR "Smart Glasses"[Mesh] OR "Imaging, Three-Dimensional"[Mesh] OR "Extended Reality"[Title/Abstract:~2] OR "Mixed reality"[Title/Abstract:~2] OR "Augmented reality"[Title/Abstract:~2] OR "Virtual reality"[Title/Abstract:~2] OR "Head mounted display"[Title/Abstract] OR "Hololens"[Title/Abstract] OR "Three dimensional imaging"[Title/Abstract] OR "3D imaging"[Title/Abstract])

AND

("Free Tissue Flaps"[Mesh] OR "Perforator Flap"[Mesh] OR "Free flap surgery"[Title/Abstract] OR "free tissue flap*"[Title/Abstract] OR "Perforator flap"[Title/Abstract:~2] OR "Free flap"[Title/Abstract:~2] OR "Microsurgical Free Flap*"[Title/Abstract] OR "DIEP flap*"[Title/Abstract] OR "deep inferior epigastric perforator flap*"[Title/Abstract] OR "ALT flap*"[Title/Abstract] OR "anterolateral thigh flap*"[Title/Abstract] OR "SCIP flap*"[Title/Abstract] OR "superficial circumflex Iliac Artery Perforator flap*"[Title/Abstract] OR "SIEA flap*"[Title/Abstract] OR "superficial inferior epigastric artery flap*"[Title/Abstract] OR "TRAM flap*"[Title/Abstract] OR "transverse rectus abdominis myocutaneous flap*"[Title/Abstract] OR "TAP flap*"[Title/Abstract] OR "thoracodorsal artery perforator flap*"[Title/Abstract])

**Ovid:**

(augmented reality/ or augmented reality system/ OR virtual reality/ or virtual reality head mounted display/ OR head-mounted display/ OR three-dimensional imaging/ OR (Extended ADJ2 Reality OR Mixed ADJ2 reality OR Augmented ADJ2 reality OR Virtual ADJ2 reality OR "Head mounted display" OR "Hololens” OR "Three dimensional imaging" OR "3D imaging").ti,ab,kf.)

AND

(free tissue graft/ or surgical flaps/ or tissue flap/ OR perforator flap/ OR ("Free flap surgery” OR “free tissue flap*” OR Perforator ADJ2 flap OR Free ADJ2 flap OR "Microsurgical Free Flap*" OR "DIEP flap*" OR "deep inferior epigastric perforator flap*" OR "ALT flap*" OR "anterolateral thigh flap*" OR "SCIP flap*" OR "superficial circumflex Iliac Artery Perforator flap*” OR "SIEA flap*" OR "superficial inferior epigastric artery flap*" OR "TRAM flap*" OR "transverse rectus abdominis myocutaneous flap*" OR "TAP flap*” OR "thoracodorsal artery perforator flap*").ti,ab,kf.)

**Web of Science:**

TS=((Extended near/2 Reality) OR (Mixed near/2 reality) OR (Augmented near/2 reality) OR (Virtual near/2 reality) OR "Head mounted display" OR "Hololens” OR "Three dimensional imaging" OR "3D imaging")

AND

TS=(“Free flap surgery" OR "free tissue flap*" OR (Perforator near/2 flap) OR (Free near/2 flap) OR "Microsurgical Free Flap*” OR "DIEP flap*" OR "deep inferior epigastric perforator flap*" OR "ALT flap*" OR "anterolateral thigh flap*" OR "SCIP flap*" OR "superficial circumflex Iliac Artery Perforator flap*" OR "SIEA flap*" OR "superficial inferior epigastric artery flap*" OR "TRAM flap*" OR "transverse rectus abdominis myocutaneous flap*" OR "TAP flap*" OR "thoracodorsal artery perforator flap*")

**Scopus:**

TITLE-ABS-KEY(("Augmented Reality" OR "Virtual Reality" OR "Smart Glasses" OR "Imaging, Three-Dimensional" OR "Extended Reality" OR "Mixed reality" OR "Head mounted display" OR "Hololens" OR "Three dimensional imaging" OR "3D imaging")

AND

("Free flap surgery" OR "free tissue flap*" OR "Perforator flap" OR "Free flap" OR "Microsurgical Free Flap*" OR "DIEP flap*" OR "deep inferior epigastric perforator flap*" OR "ALT flap*" OR "anterolateral thigh flap*" OR "SCIP flap*" OR "superficial circumflex Iliac Artery Perforator flap*" OR "SIEA flap*" OR "superficial inferior epigastric artery flap*" OR "TRAM flap*" OR "transverse rectus abdominis myocutaneous flap*" OR "TAP flap*" OR "thoracodorsal artery perforator flap*"))
